# Secondary Hemorrhagic Shock Due to Spontaneous Isolated Dissection of the Superior Mesenteric Artery Branch

**DOI:** 10.7759/cureus.60543

**Published:** 2024-05-18

**Authors:** Arlette Muneza, Dorian Colson, Kevin Silber, Roseline Petnga Fenyi

**Affiliations:** 1 Emergency Department, Centre Hospitalier Régional Sambre et Meuse (CHRSM) Université Libre de Bruxelles (ULB), Namur, BEL; 2 Emergency Department, Hôpital Civil Marie Curie, Université Libre de Bruxelles (ULB), Charleroi, BEL; 3 Emergency Department, Clinique Saint-Luc Bouge, Namur, BEL

**Keywords:** superior mesenteric artery, abdominal pain, endovascular treatment, hemorrhagic shock, isolated and spontaneous dissection

## Abstract

Spontaneous isolated dissection of the superior mesenteric artery (SIDSMA) is a rare condition, particularly when complicated by hemorrhagic shock. This case report describes the discovery of SIDSMA in an 88-year-old woman through CT angiography. The patient initially presented with acute abdominal pain, nausea, and diarrhea, which later progressed to hemorrhagic shock. After fluid resuscitation, the patient underwent successful endovascular treatment.

## Introduction

Spontaneous isolated dissection of the superior mesenteric artery (SIDSMA) is a rare condition that predominantly affects males in their fifties [1]. Clinical presentations vary from asymptomatic to acute abdomen, with rare occurrences of complications such as hemorrhagic shock. Reported risk factors include hypertension, other hemodynamic abnormalities, smoking, and genetic components [2,3]. With the wide availability of CT scanning for evaluating abdominal pain, an increasing number of cases are likely to be diagnosed and reported. Furthermore, the aging population presents challenges, including altered clinical presentations, different physiological responses, and altered compensations, which must be considered when determining eligibility for treatment. Therapeutic options vary according to severity and clinical state, ranging from monitoring to endovascular therapy or even surgery. However, due to the rarity of the condition, optimal management and follow-up strategies remain uncertain.

In this report, we present a case of SIDSMA complicated by hemorrhagic shock, successfully treated with endovascular intervention.

## Case presentation

An 88-year-old female presented to the emergency department with abdominal pain, nausea, watery diarrhea, and a sudden onset of chills two hours prior. She reported being bitten by her dog the previous day, resulting in redness at the site of the bite. Upon arrival, the patient no longer experienced abdominal pain. Her medical history included hypertension, hypercholesterolemia, hypothyroidism, long-term corticosteroid therapy for polymyalgia rheumatica, a bioprosthetic aortic valve, partial colectomy for colorectal adenocarcinoma, total radical hysterectomy, and breast cancer treated by mastectomy with adjuvant hormonal therapy. The patient complied with a daily regimen, including antihypertensive medication, an antiplatelet agent, a lipid-lowering agent, and corticosteroids, in addition to hormonotherapy.

Clinical examination revealed abdominal discomfort and a superficial wound on the distal left tibia, with redness extending to the foot. Blood tests showed a hemoglobin level of 11 g/dL, a creatinine level of 1.3 mg/dL, and lactate dehydrogenase levels of 273 U/L, but no signs of inflammation were present on the first day. Abdominal CT without contrast revealed an indeterminate inter-polar formation on the right kidney, suggestive of either a dromedary hump or a malignant structure. Microbiologic tests, including a wound swab on the tibia and blood cultures, were also conducted, and the patient was hospitalized. The following day, blood samples revealed a stable hemoglobin level, mild hyperleukocytosis (10.8 thousand/mm³) with a predominance of neutrophils (9.6 thousand/mm³), and increased inflammation (CRP 93 mg/L) (Table 1).

**Table 1:** Blood samples

Hematology	Patient day 1	Patient day 2	Reference range
Red blood cell count (RBC)	3.94	3.59	4.5-5.9 × 10^6^ cells/mm^3^ (men) 4.1-5.1 × 10^6^ cells/mm^3^(women)
Haemoglobin (Hgb)	11.7	10.7	14-18 g/dL (men) 12-16 g/dL (women)
Hematocrit (Hct)	35.8	32.3	42-50% (men) 36-45% (women)
Mean corpuscular volume (MCV)	91	90	80-96 micron3
White blood cell count (WBC)	7.4	10.8	4.0-10.0 × 10^3^ cells/mm^3^
Platelet count (Plt)	259	208	150-400 10^3^ cells/mm^3^
Inflammatory tests			
C-reactive protein (CRP)	4.08	93.9	<5 mg/L
Coagulation			
Prothrombin time (PT)	99	/	>70 %
International normalized ratio (INR)	1.0	/	<1.2
Activated thromboplastin time (aPTT)	23.4		24-36 sec
Glycemia	157	107	70-100 mg/dl
Creatinine	1.3	1.2	0.3-0.9 mg/dl
Glomerular filtration rate (GFR) (modification of diet in renal disease (MDRD) - Caucasian)	39	44	>60 ml/min
Enzymology			
Alanine aminotransferase (ALT)	28	19	10-35 U/L
Aspartate aminotransferase (AST)	18	15	10-35 U/L
Alkaline phosphatase (ALP)	83	62	30-105 IU/L
γ-Glutamyl transpeptidase	43	37	<36 U/L
Lactate dehydrogenase (LDH)	273	262	<250 U/L
Lipase	16	/	13-60 U/L
Creatinine kinase (CK)	61	45	<165U/L

**Figure 1:**
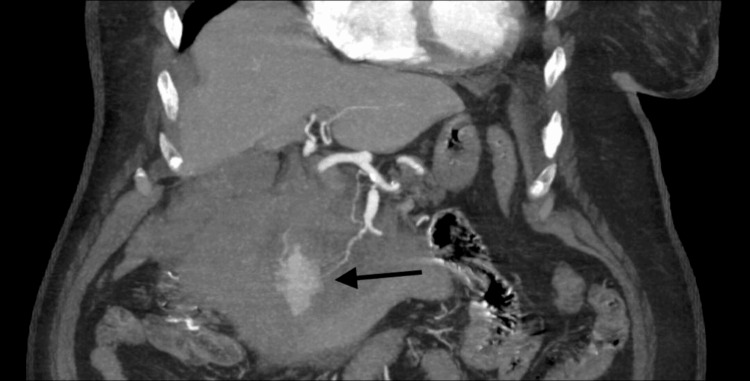
Computed tomography (CT) image demonstrating intra-abdominal hemorrhage with contrast extravasation

An abdominal ultrasound was conducted to assess the lesion of the right kidney, which was identified as a Bosniak type 1 cyst. The examination also revealed the presence of fluid in the duodenopancreatic groove, prompting further evaluation via contrast-enhanced CT. This imaging revealed a retroperitoneal hematoma worsening in real-time during image acquisition, with arterial leakage against the wall of the second part of the duodenum (Figure 1).

After the imaging procedure, the patient experienced severe hypotension but transiently improved after being placed in the Trendelenburg position with oxygen support. At that time, arterial blood gas analysis revealed a drop in hemoglobin levels (8.7 g/dL) (Table 2).

**Table 2:** Arterial blood gas

Arterial blood gas n°1 - day 2	Patient day 2	Reference range
Haemoglobin	8.7	12-16 g/dL
pH	7.39	7.35-7.45
Partial pressure of carbon dioxide (pCO2)	37	36.0-42.0 mmHg
Bicarbonate	22.4	19-21 mmol/L
Base excess	-2.2	-3.0-3.0 mmol/L
Standard bicarbonate	23.2	21-26 mmHg
Partial pressure of oxygen (pO2)	178	75-105 mmHg
Lactic acid	13.0	4.5-14.4 mg/dL

She was subsequently transferred to the ICU for stabilization before being referred to a facility equipped with interventional radiology. The patient developed hemorrhagic shock, and fluid resuscitation was initiated with albumin, crystalloids, and the transfusion of three units of O-negative packed red blood cells, along with the initiation of vasopressor support using norepinephrine (Table 3). Once the patient showed improvement, she was transferred to the nearest facility equipped with angiography.

**Table 3:** Arterial blood gas

Arterial blood gas n°2 - day 2	Patient values	Reference range
Haemoglobin	6	12-16 g/dL
pH	7.34	7.35-7.45
Partial pressure of carbon dioxide (pCO2)	34	36.0-42.0 mmHg
Bicarbonate	22.4	19-21 mmol/L
Base excess	-6.6	-3.0-3.0 mmol/L
Standard bicarbonate	19.8	21-26 mmHg
Partial pressure of oxygen (pO2)	106	75-105 mmHg
Lactic acid	40	4.5-14.4 mg/dL

**Figure 2:**
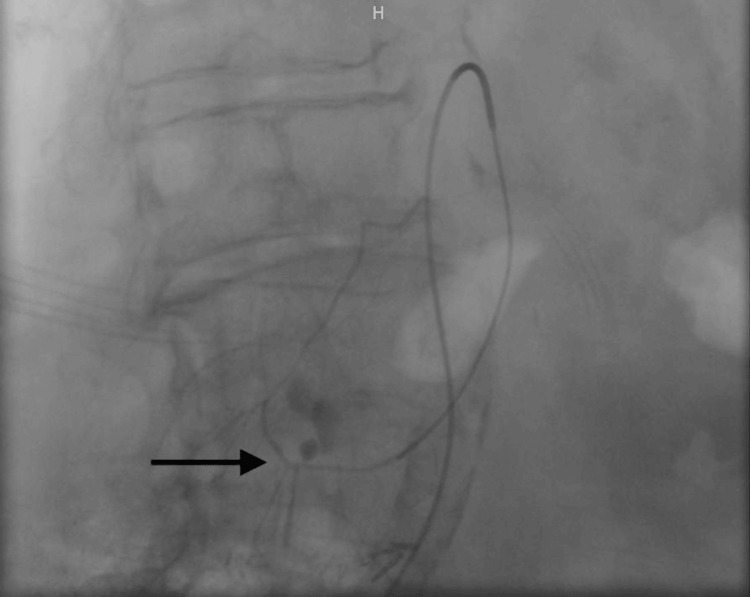
The angiogram revealed contrast leakage caused by the rupture of a dissection in a branch of the superior mesenteric artery

**Figure 3:**
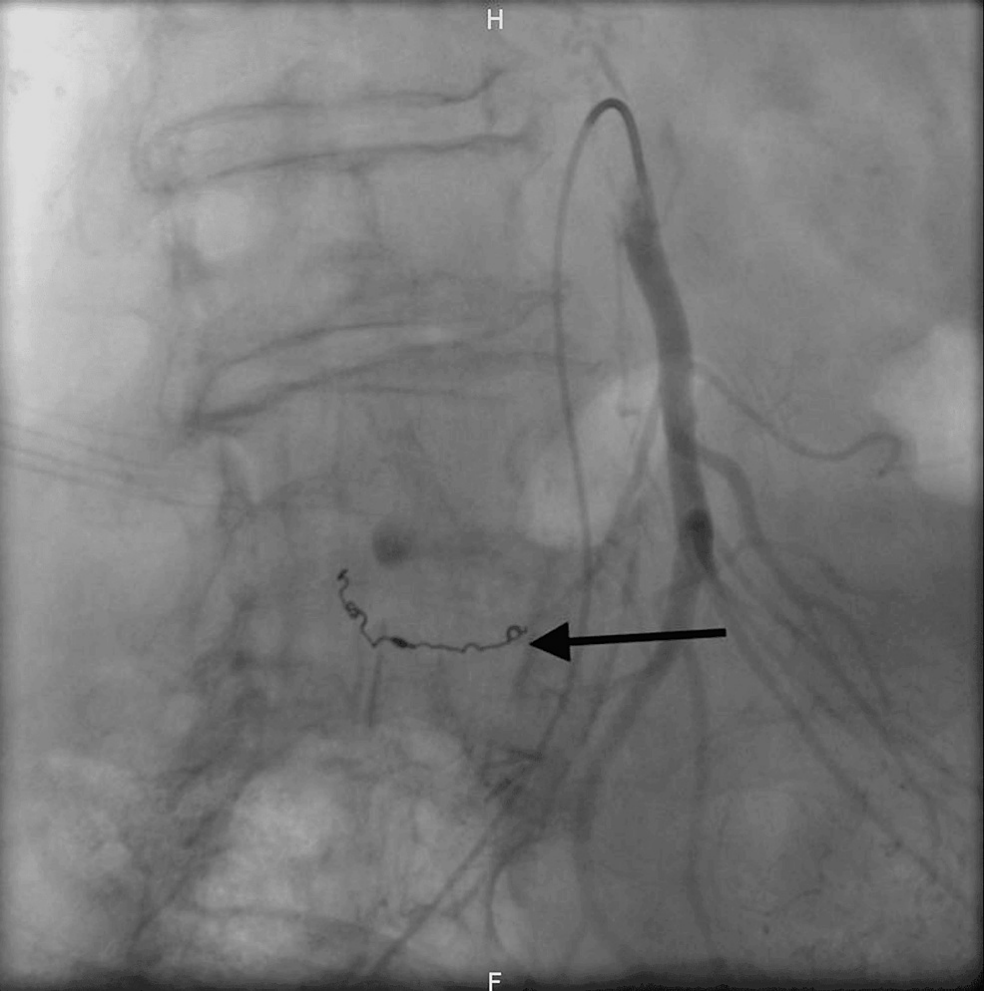
Following arterial embolization, angiography demonstrates the cessation of contrast medium extravasation

The procedure revealed a dissection of a branch of the superior mesenteric artery at the level of the second duodenum, which was treated by embolization (Figures 2-3). During the procedure, the patient required a massive transfusion protocol. Subsequently, she was transferred to the ICU. Although initial vasopressor support was necessary, the patient's condition rapidly improved, and norepinephrine was gradually tapered off within a few hours. She was able to resume oral intake on the first day after the procedure. However, she did develop bacteremia caused by *Capnocytophaga canimorsus* secondary to the dog bite, which was treated with penicillin.

The patient was discharged from the hospital after a 10-day stay in the geriatric department. A follow-up CT scan conducted four months later showed near resolution of the hemoperitoneum, with only a small subhepatic collection remaining.

## Discussion

SIDSMA is a rare and often underdiagnosed condition that primarily affects men in their fifth decade of life [1,2]. The first case of SIDSMA was described in 1947 by Bauersfel. Most reported cases have occurred in Asia, suggesting a possible genetic predisposition. Other risk factors include male gender, hypertension, and tobacco use. SIDSMA has also been associated with vascular diseases such as fibromuscular dysplasia, medial degeneration, and colorectal and gastric cancers [2]. Another frequently mentioned cofactor is an increased angle between the superior mesenteric artery and the distal aorta (>70°), which has been linked to a more symptomatic presentation [3].

Dissection, characterized by a tear in the tunica intima and internal elastic lamina resulting in the formation of a parallel false lumen, is considered isolated when the tear does not originate from the aorta or another arterial trunk and is classified as spontaneous in the absence of an identified cause. The dissection of the superior mesenteric artery typically occurs 1 to 3 cm distal to its origin, where anterior wall shear stress is high due to the curvature the artery forms, transitioning from a static retroperitoneal position to a mobile segment [2,4].

The clinical presentation varies. While over 10% of patients are asymptomatic and are incidentally diagnosed, the main symptom of SIDSMA is abdominal pain, which may or may not be preceded by lower back pain and can be acute or chronically postprandial [2,5]. Other nonspecific digestive signs, such as diarrhea and hematochezia, may also occur [6]. The clinical course can progress to hemorrhagic shock or mesenteric ischemia.

Diagnosis is made using abdominal CT angiography, which allows classification systems to guide therapeutic management. The first classification system was described by Sakamoto, who categorized SIDSMA into four types: type I features a patent false lumen without a re-entry site; type II features a patent false lumen with a re-entry site; type III features a thrombosed false lumen with an ulcer-like projection; and type IV features thrombosis of the false lumen without an ulcer-like projection [2,7]. Yun et al. later found a positive correlation between dissection length and pain severity [7].

The therapeutic approach for managing SIDSMA depends on the patient’s clinical state, and there is no consensus in the literature for its management. However, several algorithms for the management of SIDSMA have been presented. Asymptomatic or uncomplicated symptomatic cases are treated conservatively with fasting, analgesia, and antihypertensive drugs with or without antithrombotic agents, such as antiplatelet agents and anticoagulants. Katsura et al. reported that the use of anticoagulant agents could help prevent thrombus formation in a dissected SMA, while Takahashi et al. suggested the use of anticoagulants when there is a confined clot (type IV of the classification by Sakamoto) or in cases of true lumen reduction [8,9]. Ahn et al. did not advocate for the use of antithrombotics for any type of SIDSMA treated conservatively, citing a lack of demonstrated benefit on outcomes [10]. However, it's noted that conservative treatment failure can occur in up to 16% of cases [3].

Endovascular treatment may be considered if symptoms persist for seven days or more after conservative treatment, if there is a progression of the dissection on imaging, or if there is an associated pseudoaneurysm, especially if it is larger than 2 cm [2,3,11]. However, recent studies have shown that practitioners tend to opt for early endovascular treatment in all symptomatic patients, given the favorable outcomes associated with stenting, including a 99% patency rate and a 95.8% event-free survival rate at five years [12]. It is important to note that embolization can lead to complications such as distal cessation of blood flow, which fortunately did not occur in our patient's case.

Surgical treatment is reserved for complicated cases that do not respond to endovascular treatment or if the patient’s clinical condition necessitates it, such as in cases of mesenteric ischemia or vessel rupture [13].

For follow-up, CT angiography should be performed to monitor the progression or resolution of the dissection, to exclude possible complications, and to verify the patency of any stents. However, there is currently no consensus on the recommended time interval for follow-up imaging. Ultrasound (duplex and contrast-enhanced) may be an alternative for surveillance in certain cases, and it is unclear if follow-up imaging is necessary in asymptomatic cases [3].

The mortality rate for all patients with SIDSMA is low (<1%), but it can increase to 18% in patients treated surgically [2,3,14]. Currently, there is a lack of evidence regarding the management of spontaneous dissection of the upper digestive arteries due to the relative rarity of the condition.

## Conclusions

SIDSMA is indeed a rare condition, but it should be considered in the differential diagnosis of abdominal pain. CT angiography imaging plays a crucial role in diagnosing and guiding the management of patients, in conjunction with clinical findings. There is currently no consensus on the optimal management of the lesion, but possible options include conservative treatment with or without antithrombotic agents, endovascular treatment (which tends to be more commonly used as a first-line treatment for symptomatic SIDSMA), or surgery in cases of mesenteric ischemia or aneurysm rupture. To gain a better understanding of this pathology, larger-scale studies over a longer period of time would be necessary.
